# Non-contributory social transfer programs in developing countries: A new dataset and research agenda

**DOI:** 10.1016/j.dib.2017.10.066

**Published:** 2017-11-03

**Authors:** Marina Dodlova, Anna Giolbas, Jann Lay

**Affiliations:** a University of Passau, GIGA German Institute of Global and Area Studies, and CESifo Research Network, Innstr. 29, 94032 Passau, Germany; b University of Goettingen and GIGA German Institute of Global and Area Studies, Neuer Jungfernstieg 21, 20354 Hamburg, Germany

**Keywords:** Social assistance, Social transfers, Social policy design, Developing countries, New data

## Abstract

Social transfer programs in developing countries are designed to contribute to poverty reduction by increasing the income of the poor in order to ensure minimal living standards. In addition, social transfers provide a safety net for the vulnerable, who are typically not covered by contributory social security. The question of how effective such programs are in achieving these aims has been the subject of numerous impact evaluations. However, the optimal design of such programs is still unclear. Even less is known about whether the adoption and implementation of transfer programs is really driven by poverty and neediness or whether other factors also have an influence. To investigate these and other research questions, we have developed a new dataset entitled Non-Contributory Social Transfer Programs (NSTP) in Developing Countries. One advantage of this dataset is that it traces 186 non-contributory programs from 101 countries back in time and presents them in panel form for the period up until 2015. The second advantage is that it contains all the details regarding the various programs’ designs as well as information on costs and coverage in a coded format and thus facilitates both comparative quantitative and in-depth qualitative analyses. While describing the data we discuss a number of examples of how the dataset can be used to explore different issues related to social policies in developing countries. We present suggestive evidence that the adoption of social transfer programs is not based only on pro-poor motives, but rather that social policy choices differ between political regimes.

## 1 Introduction

Social protection programs can be an important instrument in fighting poverty and protecting the vulnerable. Since the beginning of the 1990s, the number of anti-poverty transfer programs in developing countries has increased significantly. At the same time, the design and efficiency of such programs remains subject to debate. The major areas of dispute involve the trade-off between current and future poverty alleviation; the selection and social exclusion problems involved in designing social protection programs; and these programs’ regularity, duration, and budget size. Numerous studies have explored the efficiency and effectiveness of such programs in different country contexts. However, to the best of our knowledge, there is little work that takes a global perspective on social transfer programs in the developing world. This paper introduces a new dataset that provides such a comprehensive overview of social transfer programs in developing countries.

Many scholars stress the gap in comparable data on social assistance for non-OECD countries [1], [2], [3]. The existing sources usually comprise data on one type of social policy or are focused mostly on one region or only on developed countries. Typically, the time span is not large and the information is available only for the most recent 10 to 15 years. One exception is the database by Barrientos et al. [4], which combines the data on social assistance for developing countries; however, it presents only the descriptive profiles of social assistance programs, which cannot be easily used in a comparative analysis and only provide coverage up until 2010. Our Non-Contributory Social Transfer Programs (NSTP) Dataset^1^ significantly extends the work by Barrientos et al. [4] in terms of both time and space. We have checked the existing information on social transfer programs and included 102 additional programs. In total, our database comprises 186 program profiles in 101 countries. What is more important is that we encode all the details and characteristics of social transfer programs in panel form so that the data can be used for any type of quantitative and qualitative analysis. We list elements of the design such as the type of transfer, type of conditions, targeting mechanism, delivery mode, donor involvement, and pilot status, as well as cost and coverage numbers where this information is available. This type of table format for the data allows for a closer look at social policies in the developing countries from a global perspective. It thus facilitates comparative analyses according to numerous characteristics of the programs. Our database is intended to be an innovative tool to study worldwide trends in social assistance, evaluate the performance of individual schemes, and identify effective and efficient features of social transfer programs.

On the one hand, the NSTP dataset can be used to examine all transfer programs in panel form in a cross-country quantitative study. On the other hand, it allows for a focus on specific program characteristics such as different types of transfers, conditions, or targeting mechanisms. Such characteristics can easily be compared across regions or countries. The dataset provides information on every program profile, which can be used for a quick search of the details of any program in operation. Thus, it can be used by qualitative scholars to identify those programs with specific characteristics for further in-depth study.

After describing the data, we briefly review the main strands of the literature on the effectiveness/efficiency of social policy and suggest how the NSTP dataset may be used to explore open questions. To provide a more specific example that demonstrates the possible applications of the data, we focus on a particular research question: To what extent is the expansion of social transfer programs in the developing world driven by factors that are not related to pro-poor motives? We consider the political basis for the adoption of social transfer programs using proxies from the Polity IV dataset [5]. We find that democracies have more social transfer programs on average. Also, democratic regimes more often adopt conditional cash transfers (CCTs). In contrast, unconditional family support programs are significantly more widespread in non-democratic regimes, and public works programs are slightly more common. Moreover, we find that non-democratic regimes employ more targeting methods that are prone to strategic misuse and lead to less objectivity in the allocation of benefits. These regimes appear to demonstrate more political than pro-poor targeting.

The paper proceeds as follows. The next section discusses the structure and sources of the new dataset. Section 3 points out possible lines of research and proposes some insights from the literature that might be examined using the new data. Section 4 presents preliminary statistics on the link between political regimes and particular program characteristics.

## 2 Data

The Non-Contributory Social Transfer Programs (NSTP) in Developing Countries Dataset aims to provide a comprehensive overview of progressive and institutionalised social transfer programs that are intended to mitigate poverty and, often, to incentivise households to invest in long-term development to escape from poverty. The programs in the database are public, non-contributory, and rolled out on a large scale at the national level. In order to capture the redistribution efforts of governments, we include only public programs and exclude private initiatives carried out by NGOs or religious entities. The focus on non-contributory programs ensures that we capture progressive redistribution. In order to be truly pro-poor, social transfer schemes need to be available to informal sector workers and hence not be tied to formal employment. We further focus on large initiatives that have the potential to have a significant impact on poverty at the national level. Pilot programs that are likely to be scaled-up to the national level have also been included. Thus, our database lists programs that make regular transfers and that help the poor to meet their day-to-day consumption needs. We exclude one-time programs for catastrophe relief, and we purposefully do not include information on contributory social insurance systems as they typically only benefit a small and privileged segment of society (or employees) in developing countries. On similar grounds we also do not include programs that are solely available to a small group of the most destitute such as the disabled, widows, orphans, specific occupational groups, or ethnicities. We exclude such narrowly targeted programs because they hardly have a poverty mitigation effect at the national level.^2^ Although we include information on the number of beneficiaries, transfer size, and cost of programs where it is available, the lack of comparable data does not allow us to have more formal inclusion and exclusion criteria (such as cut-off points that refer to the size of the programs). As we do not provide information on all elements of social security systems in developing countries, our database should not be used to assess all the contributory and non-contributory components of countries’ social policy.

As already mentioned, the existing datasets do not cover all the available information on non-contributory social schemes in non-OECD countries [1], [2], [3]. In addition, they present information only for recent years. For example, the ILO Social Security Inquiry Database [6] lists all the components of the social security system for 97 developed and developing countries. However, it comprises information on these varied social protection initiatives only for the period from 2000 to 2012. Another solid database is the World Bank Atlas of Social Protection – Indicators of Resilience and Equity (ASPIRE) [7]. It presents aggregated indicators of social protection systems’ performance and expenditure for 117 developing countries from 1998 to 2014. The information, however, is available only for program categories, not for individual schemes. The United Nations Economic Commission for Latin America and the Caribbean (ECLAC) provides a database on non-contributory social protection programs in 22 countries within one region only: Latin America and the Caribbean [8]. Similarly, the Social Protection Index (SPI) of the Asian Development Bank compiles indices of aggregate social protection indicators for 42 countries in the Asian region for 2000 to 2010 [9]. Regarding non-contributory pension schemes, Pension Watch provides a large Social Pensions Database for 107 developed and developing countries [10]. The only comprehensive data on social assistance in developing countries is the data provided by Barrientos et al. [4]. However, they focus more on the program profiles and case study analyses, thereby disregarding a potential quantitative comparative perspective. These different data sources feed into our database, where they are complemented by further typically program-specific sources such as program evaluation reports and national social security boards. We have screened all of these and other sources to compile comparable information on non-contributory, large-scale and pro-poor transfers that can be used for both qualitative and quantitative analyses of all developing countries.

Our data collection effort extends the work by Barrientos et al. [4]. This earlier database included information on 110 social transfer programs in 55 countries up until 2010. We updated 84 of the earlier program profiles and decided not to include 26 programs because they either had been discontinued or did not meet our aforementioned criteria for inclusion. In addition, we collected new information on 102 social transfer programs that were not reported by Barrientos et al. [4]. As a result, we present 186 program profiles from 101 countries, covering the time up to 2015. We provide the data in two formats: a list and a table format. The list consists of descriptive program profiles that provide information on program characteristics and include further links to relevant program impact evaluations in the literature. The table component of the database includes both country-year and program-period panels with encoded information on program design, costs, coverage, and other elements from the descriptive program files. Thus, the NSTP dataset is organised so as to facilitate both quantitative and qualitative research.

**Fig. 1:**
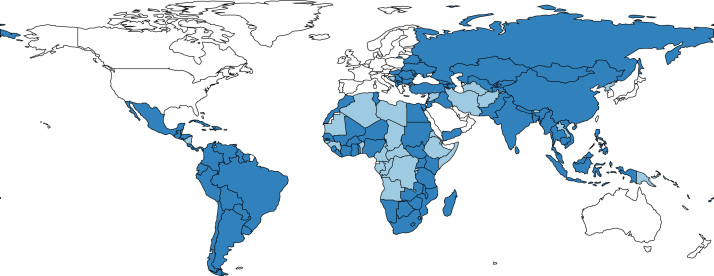
Social Transfer Programs Worldwide in 2015. *Note:* Countries with at least one transfer program are coloured dark blue (101 countries in total or 70 per cent of all developing countries).

Fig. 1 presents all developing countries that had at least one social transfer program in 2015 in dark blue. This corresponds to 101 countries in total or 70 per cent of developing countries. All developing countries that do not have a program are coloured light blue, while all developed countries are left white. We can see that while almost all countries in Latin America, Europe, and Central Asia have at least one transfer program, there are clusters of countries in Africa and the Middle East that do not have any transfer programs.

The share of countries per region with a program are as follows: 91 per cent in Europe and Central Asia, 90 per cent in Latin America and the Caribbean, 80 per cent in East Asia and the Pacific,^3^ 75 per cent in South Asia, 66 per cent in sub-Saharan Africa, and 54 per cent in the Middle East and North Africa.

Of the countries in our dataset, 55 have more than one social transfer program. Bangladesh, with eight programs, has the highest number of individual schemes in operation. However, having a larger number of transfer programs in operation does not necessarily imply broader coverage or greater spending on social assistance. In what follows, we describe the main features of the design of social transfer programs and present the variables that we code on the basis of these features. In particular, we discuss the typology of transfers and conditions, the targeting mechanisms used for beneficiary selection, and cost and coverage details. We also review the modes of delivery, donor involvement, and the status of programs (pilot or not). Other program details and characteristics, such as transfer volume or detailed eligibility criteria, which are not easily comparable across countries, are presented only in the descriptive part of the database. We refer those scholars who wish to use this information to the qualitative program profiles.

### 2.1 Typology of transfers and conditions

We distinguish between unconditional and conditional transfers. The important difference is that the recipient of unconditional transfers does not have to comply with any conditions to receive the transfer apart from meeting the targeting criteria. The beneficiary of conditional transfers has to make some kind of effort to receive the transfer, meaning that he or she usually has to comply with certain rules or types of behaviour. Of the 186 programs in the dataset, 101 are unconditional, 78 are conditional, and 7 combine elements of both conditional and unconditional schemes.

We further categorise transfers into unconditional family support schemes, social pension schemes, CCTs, and public works programs. Under unconditional family support schemes, we subsume transfers targeted to low-income households or specifically to children that are not tied to school attendance or regular health check-ups. They range from a basic safety net for those below the poverty line to (universal) child support grants. We define social pension schemes as transfers to the elderly that are independent of a history of contributions by the beneficiary or his/her employer. CCTs are programs that link the receipt of a transfer to investments in education and/or health. Health conditions usually aim to improve child and/or maternal health. Panama, however, has an old-age pension scheme that is paid conditional on regular health check-ups. Education conditions predominantly aim at improving the school enrolment and achievements of children from low-income households. Some CCTs specifically target girls or young adults. We provide information on whether the receipt of the benefit is conditional upon household investments in health, education, or both. Public works programs give out transfers in exchange for work at public employment sites. “Below market” or minimum wages are supposed to ensure that only the needy self-select into these programs. Table 1 shows all possible combinations of transfer types with percentages in brackets. The dataset includes information on 70 unconditional family support programs, 64 CCTs, 43 social pensions, and 23 public works programs. Of these programs, 14 are combinations of two types. For example, the Social Cash Transfer Program in Malawi provides unconditional cash transfers to households living in poverty and an additional benefit for each child attending school. It is hence coded as both an unconditional family support scheme and as a CCT. Of all the CCTs, 23 require an education investment and 8 a health investment; 33 are conditional upon investments in both education and health.

**Table 1:** Types of social transfer programs.

	Unconditional family support	Social pension	CCT	Public works
Unconditional family support	60 (32.26%)			
Social pension	4 (2.15%)	37 (19.89)		
CCT	3 (1.61%)	2 (1.08%)	57 (30.65%)	
Public works	3 (1.61%)		2 (1.08)	18 (9.68%)

**Table 2:** Frequency of targeting methods in 2015.

	Categorical	Geographical	Means test	Proxy means test	Community-based	Self-targeting
Categorical	39 (20.97%)					
Geographical	15 (8.06%)					
Means test	28 (15.05%)		13 (6.99%)			
Proxy means test	14 (7.53%)	3 (1.61%)		13 (6.99%)		
Community-based	8 (4.3%)	6 (3.23%)	3 (1.61%)	3 (1.61%)	5 (2.69%)	
Self-targeting	2 (1.08%)	7 (3.76%)				4 (2.15%)

*Note:* In total Table 2 includes 163 (88%) programs, whereas the remaining 23 (12%) programs use a combination of three or more targeting mechanisms. In total, 124 programs (66%) use categorical targeting, 54 (29%) use geographical targeting, 48 (26%) use means tests, 48 (26%) use proxy means tests, 35 (19%) use community-based targeting, and 15 (8%) are self-targeted.

### 2.2 Targeting

Another characteristic of social transfer programs is the targeting mechanism used to determine eligibility for a program. We follow the classifications by Barrientos [11] and Coady et al. [12] and distinguish between six types of targeting – namely, categorical, geographical, means tests, proxy means tests, community-based targeting, and self-targeting.

The simplest mechanism is *categorical targeting* based on categories defined ex ante. Benefits are given conditional on belonging to a certain age group, gender, or social category – for example, the elderly, children, women-headed households, etc. If categorical targeting is employed without any additional targeting mechanism, the transfers are in effect universal instead of poverty targeted.

A special form of categorical targeting is based on *geographical location*. In particular, the transfers are allocated to the regions identified as the poorest within a country using one or several indicators associated with a high level of poverty – for example, literacy rates, nutritional status, or consumption measures. Eligibility for a program is dependent on residence in these areas. While we do not include the transfer programs of federal states (or other decentralised governing units), we do include programs that are allocated to districts or regions defined as the poorest by the central government.

*Means testing* refers to a form of targeting that involves the assessment of the income of a household or individual by an official. If the income falls below some cut-off level, the individual or household becomes eligible for program benefits. Ideally, this implies the existence of documentable and verifiable information on income in the form of tax records, wage information from employers, or financial information from banks. However, in contexts of weak administrative capacity and/or a high share of informal labour, documenting and verifying income is not straightforward. Hence, there are large differences in the complexity and accuracy of means tests. In some cases, an officer assesses the income of a potential beneficiary in their home; in other cases the applicant is interviewed in an office with the information taken at face value.

*Proxy means tests* are similar to means tests, but instead of using information on income, they use information on observable household characteristics that are strongly correlated with poverty to calculate a score for the households’ economic situation. The information typically collected for proxy means tests in poor countries includes the quality of the dwelling, the ownership of durable goods, household composition, education level, and occupational sector. The score is then used to determine eligibility for benefits.

In *community-based targeting*, a group of community members or a community leader decides on eligibility for a program. This targeting method takes advantage of social capital – that is, the fact that local actors have more information available or at a lower cost than program officials.

*Self-targeted* programs are in principle open to all but use strong incentives to discourage use by the non-poor. Public works programs that use self-targeting based on a work requirement typically pay wages that are below the market wage for unskilled labour or the minimum wage. The low wages ensure that only the really needy self-select into the program. However, when the number of applicants exceeds the number of jobs in the program, additional targeting or selection methods need to be implemented (e.g. means tests or proxy means tests). In the latter case, the program is no longer self-selected.

Many of the programs in our sample use more than one targeting method. In fact, only 40 per cent of all programs employ a single targeting method. We therefore define a binary indicator for every targeting mechanism. Table 1 shows the frequency of targeting choices across all programs in 2015 with the percentages in brackets. In addition to the combinations displayed, approximately 12 per cent of all programs apply a combination of three or more targeting methods. The most frequent choices of targeting methods are categorical criteria only, a combination of categorical criteria with a means test, geographical criteria, or a proxy means test.

### 2.3 Cost and coverage

The most important characteristics of social assistance programs are their budget and coverage – that is, how expensive they are and how many beneficiaries they have. Along with effective and efficient targeting, the budget and coverage of social programs are principal components that contribute to structural changes in inequality and poverty levels. We report only the original source data and only if the year of the respective coverage or budget information is indicated by the source.

Depending on the program, coverage is measured in terms of individuals or households or both. We provide information on coverage of individuals for 110 social transfer programs and coverage of households for 55 programs.

We report program budget data according to two dimensions, depending on availability. The first dimension is the absolute value of program costs in either USD million or the local currency. If the program budget is presented in the local currency, we assume that these costs are in the current prices for the year as provided in the source. If the USD measure for the program budget is presented, we assume that local currency costs have been transformed into USD using the current exchange rate for the year of the source. The second dimension is the budget as a share of the country's GDP. The database includes information on the cost in USD million of 54 programs and on the cost as a percentage of GDP of 47 programs. Hence, our indicators of the cost and coverage of the programs are encoded according to the availability of data on the different measures.

### 2.4 Other elements of the programs’ design

#### 2.4.1 Delivery

The benefits provided by social transfer programs are predominantly distributed in cash. Of all the programs in our database, 155 (84 per cent) give out cash only. Cash in combination with other services such as trainings is provided by 21 programs (11 per cent). Public works programs are also counted as being among these programs. Six programs (3 per cent) give out cash in combination with food. Only four programs (2 per cent) are pure food transfers.

#### 2.4.2 Donors

Since the 1990s the expansion of social transfer programs has been actively promoted by international donors [13]. In 2015 at least one donor was involved in more than 26 per cent of programs. The donors contribute to both program funding and program design and implementation. The most influential donor is the World Bank, which supports 30 programs, followed by UNICEF (11 programs), DFID UK^4^ (11 programs), and the World Food Program (5 programs).

#### 2.4.3 Pilots

The database captures information on nine social transfer programs that were being piloted in 2015. We have also coded the years in which now-large-scale programs were pilots.

## 3 Research agenda

In the following, we briefly review some of the main strands of the literature on the effectiveness and efficiency of social policy in developing countries, highlighting gaps that could be addressed using the NSTP data. We then provide examples of how the data can be used to examine the political motivations behind the adoption of transfer programs.

### 3.1 Effectiveness and efficiency

3.1

**Fig. 2:**
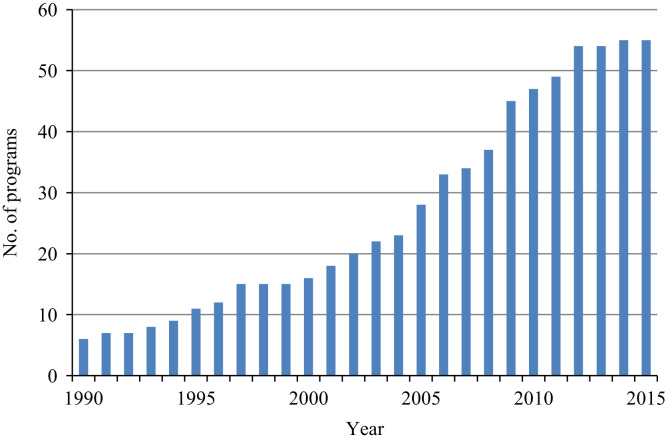
Number of social transfer programs in LAC, 1990–2015.

Fig. 2 illustrates the increase in the adoption of transfer programs in the Latin American and Caribbean (LAC) region. While there were six social transfer programs in 1990, the number had risen to 47 in 2010 and 55 in 2015. Other regions demonstrate similar patterns. This makes it evident that social policy diffusion plays a major role in poverty reduction [3], [14]. However, research in this area is still scarce and little is known about policy simulation patterns in developing countries.

Scholarly interest in transfer programs has risen accordingly in recent decades, resulting in a literature that is quite broad and interdisciplinary. One strand centres on questions related to the conceptualisation, design, and implementation of social policy. The scholars consider the methodological and theoretical aspects, with the debates focused particularly on selection and social exclusion problems, the types of transfers, program scale, and other technicalities [11], [15], [16]. The classical questions relate to the efficiency and effectiveness of unconditional versus conditional transfers, the different targeting methods, and graduation out of transfer schemes. There is strong evidence in the empirical literature that both unconditional and conditional transfers have a poverty-reduction effect. However, the debates on specific design elements of the programs that contribute most to poverty alleviation are ongoing [17]. Regarding the effect of cash transfers on school attendance, there seems to be agreement that CCTs with explicit education conditions and penalties for non-compliance have a stronger effect than unconditional transfers [18], [19]. Studies that focus on CCTs provide evidence of increased health service use and improved health outcomes [20], [21]. And recent studies that compare conditional and unconditional transfers suggest that health conditions do indeed matter [22], [23].

A few studies have recently reviewed the targeting effectiveness of social transfer programs. Coady et al. [12] find that no single targeting mechanism performs best in all contexts. Devereux at al. [24] confirm that all the targeting mechanisms generate inclusion/exclusion errors and costs and they also conclude that the appropriate choice of targeting mechanism is context-specific. At the same time, Devereux et al. [25] present evidence that community-based programs might be quite efficient and proxy-means tests are better in certain cases than categorical indicators. There are also debates on the technical part of targeting. For example, Coady and Skoufias [26] suggest an alternative approach to evaluate targeting mechanisms by decomposing their effects into two, targeting efficiency of the instrument and redistributive efficiency. Azevedo and Robles [27] conclude that a multidimensional targeting approach should be applied in order to increase the efficiency of conditional cash transfers. The NSTP data allows conducting further research on the comparison and effectiveness of targeting methods.

Not surprisingly due to the complexity of the relationships, the evidence is weakest for a positive effect of social transfer programs on social inclusion and economic growth [28]. The NSTP data could, for example, be used to analyse the link between (certain types or design characteristics of) social transfer programs and human development outcomes. In addition, the NSTP data can contribute to measuring income and redistribution; especially in the regions with poor national statistics (see for a discussion [29]). Women's empowerment is another research topic, for which the NSTP dataset can provide interesting statistics for cross-country perspective. Duflo [30] reviews that women's empowerment is closely linked to human capital accumulation and self-sustainable development.

Regarding the affordability of social assistance, one strand of literature stresses a moral argument for assisting the poor and reducing risk by providing a minimum safety net [31], [32], [33]. Another line of research focuses on modelling the cost of basic social protection [34], [35]. The third perspective on affordability concerns the sources of finance [36], [37]. This debate also centres on whether and how people working in the informal sector can be made to contribute financially to social protection [38], [39]. Further questions include the political acceptance of certain types of assistance [20], [40] and the labour market effects of extensions to social security [41], [42]. An interesting application of the NSTP data could therefore be to examine the effects of the adoption of (specific types of) social transfer programs on labour supply or the productive capacity of the poor.

### 3.2 The politics of pro-poor policies

Another important part of the literature is the research on the politics of social assistance. In this emerging subfield the main questions involve how social transfer programs promoted by international donors contribute to building state capacity and how the design and implementation of such programs are eroded by corruption, clientelism, and other political motives. Indeed, a number of interesting insights emerge from the analysis of the motivations for adopting social transfer programs in developing countries. The recent studies show that social transfer programs are not chosen primarily because of poverty reduction but are also driven by other mechanisms not related to pro-poor motives [40], [43], [44]. In particular, political leaders may use social policy in order to strengthen their rule. In democratic regimes, social benefits can be a tool to gain or reward voters [45], [46], [47], [48]. Autocracies may use transfers to mitigate social unrest by increasing the standard of living of the poor or they may channel benefits to their supporters [49], [50], [51]. There is an emerging literature on how social transfers decrease non-electoral forms of political participation such as protests and demonstration attendance [52]. In addition, leaders in both regime types may enact social policies as a response to pressure from international donors or neighbouring countries [14], [53], [54], [55].

**Fig. 3:**
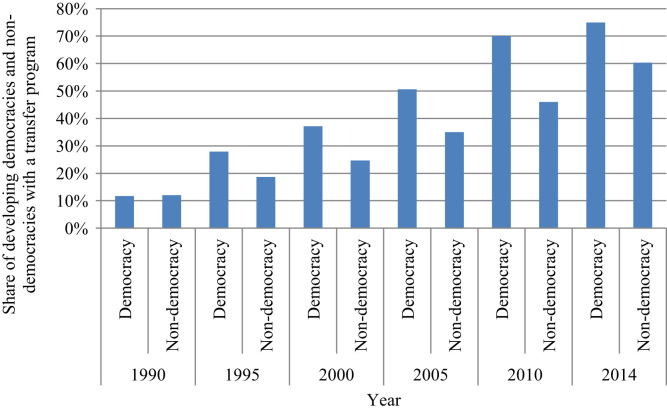
Share of developing democracies and non-democracies with a transfer program, 1990–2014. *Note:* The data on the polity score extends until 2014.

In what follows, we use the NSTP dataset to provide suggestive evidence on the political economy of social transfer programs. We consider whether political motives or institutions affect the design of transfer programs. Political regimes particularly influence the scope and structure of social policy. Hence, we focus on additional factors not related to purely pro-poor motives that shape social policy in developing countries. First, we explore the prevalence of transfer programs in democracies versus non-democracies. Fig. 3 shows the percentage of developing democracies and non-democracies^5^ that had at least one transfer program between 1990 and 2014 in five-year intervals. Of all the developing countries, 12 per cent of democracies and 12 per cent of non-democracies had a transfer program in 1990. We see that starting in the mid-1990s, the share of countries with at least one social transfer program increased steadily in all regime types, though significantly more in the case of democracies. In 2014, 75 per cent of countries classified as democracies and only 60 per cent of countries classified as non-democracies had at least one transfer program.

Of the 162 programs for which we have information on the polity type in the year of adoption of a program, 81 (50 per cent) were adopted by democratic countries, 58 (36 per cent) were adopted by anocracies, and 23 (14 per cent) by autocracies.

**Fig. 4:**
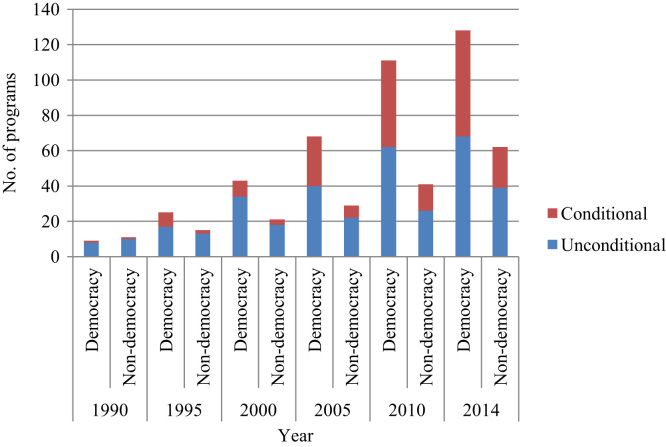
Transfer program types in democracies and non-democracies, 1990–2014. *Note:* The data on the polity score extends until 2014.

Second, we explore systematic differences in the types of transfer programs according to regime type. Fig. 4 shows the increase in the number of unconditional and conditional transfer programs between 1990 and 2014 in democracies and non-democracies in five-year intervals. We see that starting from the mid-1990s, the number of both types of programs increased steadily in both regime types. In total, more transfer programs were adopted in democracies, with the total number in 2014 being roughly twice the number of programs in non-democracies (128 versus 62).^6^ Moreover, in democracies more conditional programs were adopted than in non-democracies. In 2014, democracies had 60 (47 per cent) conditional programs and 68 (53 per cent) unconditional programs, while non-democracies had 23 (37 per cent) conditional programs and 39 (63 per cent) unconditional ones. Regarding the subcategories of programs in 2014, democracies had 40 unconditional family support programs (30 per cent), 30 pension schemes (23 per cent), 47 CCTs (36 per cent), and 14 public works programs (11 per cent). Non-democracies had 28 unconditional family support programs (45 per cent), 11 pension schemes (19 per cent), 14 CCTs (22 per cent), and 8 public works programs (13 per cent).

There thus appears to be a correlation between a higher score on the polity scale and having a social transfer program. This is in line with the literature on the link between regime type and redistribution, according to which democratic countries are more likely to have social transfer programs [43], [56]. Moreover, we see that democracies apply more programs with human capital investments. This is very probably connected to the fact that democracies care more about the long-term developmental effects of pro-poor policies [57]. We can assume that non-democracies are interested in more unconditional transfer programs because the latter provide faster short-term effects, which help regimes to sustain power and decrease civil unrest in a society.

**Fig. 5:**
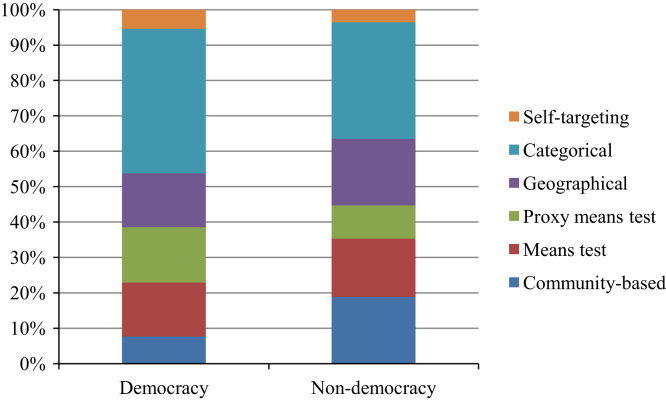
Targeting mechanisms by regime type in 2014. *Note*: The data on the polity score extends until 2014.

Finally, we are interested in the choice of targeting mechanisms, and specifically their potential to be used for political reasons in different regime types. It appears that programs with a certain type of targeting are promoted more in non-democracies because they may be more easily manipulated in the interest of local elites or politicians. Fig. 5 shows the share of each targeting method by regime type in 2014. We see that geographical targeting is used by 19 per cent of programs in non-democracies and 15 per cent in democracies. Community-based targeting is also more prominent in non-democracies: 19 per cent of programs there use this method versus 8 per cent of programs in democracies. Proxy means tests are used more frequently in democracies, where they have a share of 15 per cent as opposed to a share of 9 per cent in non-democracies. Categorical targeting is also applied more in democracies, with this method used by 41 per cent of programs versus 32 per cent in non-democracies. Means tests and self-targeting are equally present in both regime types and represent approximately 15 and 5 per cent of all programs, respectively. These shares indicate systematic differences in the choice of targeting mechanisms between regime types.

As already mentioned, two targeting mechanisms are particularly dominant in non-democracies: community-based targeting and geographical targeting. When beneficiary selection is undertaken by a third party, it can be expected that this third party will act according to motives that are not in line with providing the most accurate pro-poor targeting. As a result, a possible explanation for why community-based programs are applied more often in non-democracies is that they leave room for subjective or politically motivated decisions in the allocation of benefits [58]. Local leaders/elites have a greater degree of discretion and their subjective considerations may impact the selection of beneficiaries into a program. The rent-seeking and clientelistic motives of community leaders may distort the efficiency of such targeting in non-democracies, while also making this type of targeting more attractive. Moreover, this form of targeting can perpetuate local power structures, and certain minorities can be systematically excluded. Geographical targeting is likely to be dominant in non-democracies because the incumbent leaders/parties can use it to reward loyal districts or, on the contrary, avoid social unrest in certain districts (strongholds versus swing voters). Especially in combination with other targeting mechanisms, geographical targeting may become more political than pro-poor. From our perspective, other interesting applications of the NSTP data could include analyses of the diffusion of (certain types of) social transfer programs across regions or the relationship between transfer programs and state capacity.

## Footnotes

1 Data is freely available in the GESIS Data Archive. http://dx.doi.org/10.7802/1530

2 An exception to this rule is orphan care programs in countries with a high prevalence of HIV/AIDS where there is a large number of orphans. Information on pensions for the disabled and widows is included where the former are a part of general non-contributory old-age pensions.

3 This share is calculated excluding small island states.

4 Department for International Development – United Kingdom.

5 We use the polity score from the Polity IV Project [5] to distinguish between polity types. The polity score classifies countries on a scale of -10 to 10. Countries with a score above 5 are classified as democracies, countries with a polity score between -5 and 5 are classified as anocracies, and countries with a polity score below -5 are classified as autocracies. Information on the polity score only extends until 2014.

6 The figure includes the seven programs that are coded as both conditional and unconditional. Hence, the numbers given here exceed the total number of 182 programs in 2014.
